# A Machine Learning Approach to Growth Direction Finding for Automated Planting of Bulbous Plants

**DOI:** 10.1038/s41598-019-57405-8

**Published:** 2020-01-20

**Authors:** Brian G. Booth, Jan Sijbers, Jan De Beenhouwer

**Affiliations:** Imec-Vision Lab, Department of Physics, University of Antwerp, B-2610 Antwerp, Belgium

**Keywords:** Computational science, Software, Data processing, Image processing, Machine learning

## Abstract

In agricultural robotics, a unique challenge exists in the automated planting of bulbous plants: the estimation of the bulb’s growth direction. To date, no existing work addresses this challenge. Therefore, we propose the first robotic vision framework for the estimation of a plant bulb’s growth direction. The framework takes as input three x-ray images of the bulb and extracts shape, edge, and texture features from each image. These features are then fed into a machine learning regression algorithm in order to predict the 2D projection of the bulb’s growth direction. Using the x-ray system’s geometry, these 2D estimates are then mapped to the 3D world coordinate space, where a filtering on the estimate’s variance is used to determine whether the estimate is reliable. We applied our algorithm on 27,200 x-ray simulations from *T. Apeldoorn* bulbs on a standard desktop workstation. Results indicate that our machine learning framework is fast enough to meet industry standards (<0.1 seconds per bulb) while providing acceptable accuracy (e.g. error < 30° in 98.40% of cases using an artificial 3-layer neural network). The high success rates of the proposed framework indicate that it is worthwhile to proceed with the development and testing of a physical prototype of a robotic bulb planting system.

## Introduction

In recent years, agricultural robotics has become an area of intense research as governments around the world target the economic potential of automation in this sector^1,2^. These economic advantages stem not only from reduced labour costs, but also from more precision planting, harvesting, and advanced care of crops, leading to higher yields^3^. These reduced production costs can then help in reducing food costs for consumers. The flower growing industry have also shown interest in robotic agricultural techniques in an attempt to produce higher and more consistent yields^4^.

Robotic techniques have been applied to a variety of agricultural tasks, from seeding and transplanting^5,6^, to weeding^7^, to harvesting^8^. In each of these tasks, agricultural robots need the ability to identify the objects they wish to manipulate and be able to perform the manipulation without damaging the object^9^. These needs have led to a variety of computer vision techniques for object detection^10–12^ and obstacle avoidance^13,14^. The computer vision algorithms developed in this field vary widely depending on the plants and tasks in question, highlighting the need for specific solutions to different agricultural robotics problems.

One such example of an agricultural robotics task is the automated planting of bulbous plants like onions, garlic, and flower bulbs. This task shares similarities with seeding and transplanting, but differs from both in one key aspect: a plant bulb has a clear direction of growth that is unknown to the robot^15^. Given the desire to optimize plant height, crop yield, and crop uniformity, plant bulbs should be planted with their growth directions pointed vertically upward^16–18^. Positioning the bulb in this orientation reduces the amount of energy the plant has to expend in order to sprout above the ground, resulting in larger, more consistent yields^19–21^. This constraint on bulb orientation is not present with traditional seeding tasks where the seed can be inserted into the ground in any pose. This task also differs from transplanting where the plant has already been planted and, therefore, its growth direction is already determined. Effectively, the planting of bulbous plants constitutes its own unique task and requires its own computer vision algorithm, one that can identify a plant bulb’s growth direction.

To the best of our knowledge, no algorithm currently exists that can consistently identify the growth direction of a plant bulb, despite the noted desire for such an algorithm to be created^19,22^. Aksenov *et al*. recently proposed the first oriented planting robot that aims to grip the tip of a plant bulb’s in order to position it appropriately during planting, however their approach only achieves a successful bulb planting 51% of the time^16^. These results indicate that a plant bulb’s tip is not consistently a prominent feature. The bulb’s shape, or the presence of attached bulblets, can make it difficult to identify the plant bulb’s tip by shape alone. In an attempt to motivate research on this problem, a set of minimum criteria for such an algorithm have been published^22^. We specifically note that these criteria include (a) the planting of 10 plant bulbs per second, and (b) the planting of bulbs so that their growth directions are at least within 30 degrees of the upward vertical direction.

We hypothesize that multiple visual and morphological features are required from plant bulbs in order to estimate their growth directions in a way that satisfies the above criteria. Plant bulbs are known to have a complex internal structure that includes a root base, a central shaft from which the bulb sprouts, and shell-shaped scale surrounding the shaft that provide the plant with nutrients^15^. These internal structures provide a wealth of information on the bulb’s growth direction and we aim to incorporate these structures into a growth direction estimation algorithm. X-ray imaging has the ability to visualize these information-rich internal structures, making it an appealing imaging technique for the growth direction estimation task. X-ray imaging has also been applied to similar agricultural tasks from seeding^23^ to food quality assessment^24–26^, suggesting that it can be safely and effectively used for our task of plant bulb growth direction finding.

**Figure 1:**
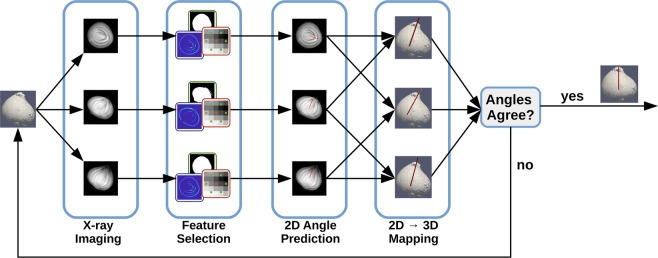
Flow chart of the proposed growth direction finding algorithm. Three x-ray images of the flower bulbs are processed to identify key shape, edge, and texture features. Machine learning algorithms then use these features in a regression to predict the growth direction within each 2D image. These 2D estimates are then paired and mapped to 3D to create three 3D estimates. If these estimates agree, then their average is outputted. Otherwise, the flower bulb is imaged again. See text for further details.

In this paper, we introduce – to our knowledge – the first algorithm for the estimation of plant bulb growth directions. An overview of the algorithm is presented in Fig. 1. The algorithm takes as input 3 x-ray projections of a plant bulb from which shape^27^, edge^28,29^, and texture features^30,31^ of the bulb are extracted. These features are then used as input to machine learning regression algorithms^32–34^ in order to estimate the 2D growth direction of each bulb in each x-ray projection. These 2D estimates of the bulb’s growth direction, one from each of the three x-ray projections, are then paired, and by taking into account the x-ray source and detector geometry, three 3D estimates of the bulb’s growth direction are computed^35^. The mean of these 3D estimates is then used as the final growth direction estimate, while the variance of these estimates is used to gauge the algorithm’s confidence in the final result^36^. Estimates with low confidence are ignored and the corresponding plant bulb is imaged a second time.

Our algorithm shows similarities to others seen in x-ray luggage screening and defect detection in manufactured parts^37^. In both contexts, object features are extracted from multiple x-ray images and are used to classify the image as either passing or failing inspection^38–42^. The choice of image features is often dependent on the application, though a thorough review of image feature detectors notes that detectors that incorporate orientation and scale perform particularly well in general^43^. Meanwhile, the choice of classifier is often dependent on the complexity of the classification and on the amount of data available to train the algorithm. In x-ray screening tasks, popular choices have included thresholding^42^, support vector machines^39,40^, and artificial neural networks^41^. In some cases, image features are used to identify outliers and the presence of a single outlier leads to a failed x-ray inspection^38^. Our research problem differs from the ones in these works as we do not have a classification problem but a regression problem: we aim to predict the growth direction from x-ray image features.

We evaluate the proposed algorithm on 81,600 x-ray projections randomly simulated from computed tomography (CT) scans of 68 *T. Apeldoorn* flower bulbs. These x-ray simulations are combined into groups of three to produce 27,200 inputs to our algorithm (400 per flower bulb). The algorithm’s training and testing is then performed in a leave-one-plant-bulb-out fashion. The results of these tests are then compared to industry-established standards for this growth direction finding task^22^.

**Figure 2:**
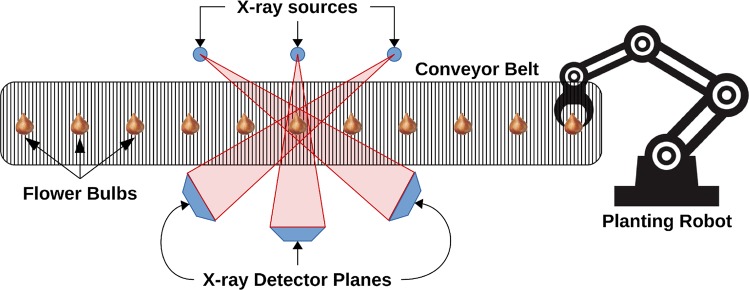
Schematic of the imaging setup simulated in this study: flower bulbs pass along a conveyor belt in the path of three x-ray projection systems before being planted by a robot. The x-ray systems are separated by 60° in order to give unique views of the same bulb.

To generate the simulated x-ray projections, we assume they are obtained from the scenario shown in Fig. 2, one which is similar to that described by Thompson *et al*.^44^. Effectively, we envision each plant bulb being transported along a conveyor belt in a uniformly-likely random pose. As the bulb travels along the belt, it will be imaged by three x-ray projection systems positioned at the sides of the belt, at the same height, and fanned out at angles 60° apart from each other. At the end of the conveyor belt will be a gripping robot that will grab the bulb, rotate it into position, and properly plant it in a growing tray^9,22^. Our objective is to estimate the necessary rotation of the plant bulbs from the x-ray projections while the plant bulbs travel along the conveyor belt.

## Results

### Data collection & system implementation

Sixty-eight bulbs of the *T. Apeldoorn* flower were scanned in sets of 9 bulbs using the large field of view Hector CT scanner at Ghent University^45^. The Hector CT scanner is equipped with a 240 kV X-ray tube (X-RAY WorX, Garbsen, Germany) and a 40 × 40 cm^2^ flat panel detector (PerkinElmer 1620, Waltham, Mass., USA) that are jointly combined on a high-precision rotation stage. The resolution of the resulting CT scans was $0.35\times 0.35\times 0.35\,{{\rm{mm}}}^{3}$. During scanning, the plant bulbs were placed into styrofoam holders in order to keep them separated and stabilized during the imaging procedure. Each of the 68 flower bulbs were then manually segmented from the styrofoam background in the CT images using a combination of thresholding and morphological operations. Once segmented, a 3D mesh was generated by applying a contour filter to the bulb’s segmented CT volume, and an expert manually annotated a vector along the growth direction twice for each bulb. The mean intra-expert annotation error was found to be 1.07°.

For each plant bulb, x-ray projections were generated by reprojecting the CT images using the ASTRA toolbox^46^. The corresponding 3D annotated growth vector was also projected onto each generated x-ray image. The x-ray projections were generated to match the simulation environment shown in Fig. 2 with the bulb placed in a uniformly-chosen random orientation. Kernel support vector regression (SVR)^32^, kernel extreme learning machines (ELM)^34^, and a 3-layer fully-connected artificial neural network (ANN)^33^ were evaluated for the growth direction prediction. The proposed algorithm was implemented in MATLAB, version 2018a (The MathWorks, Natuck, USA) running on a CentOS 7 operating system. All experiments were run on a desktop workstation with 16 Intel i7-5960X processor cores running at 3.00 GHz. We initially show results from the 3 machine learning algorithms’ predictions of the 2D growth directions, one per digitally-reprojected radiograph. Subsequently, we display 3D growth direction results for the algorithm as a whole. Due to the randomness in the ELM algorithm, we show its aggregate results from 20 runs.

### Machine learning regression

**Figure 3:**
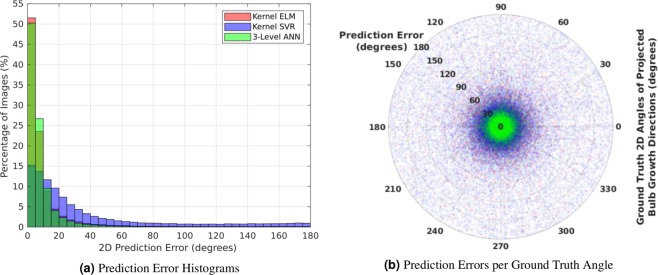
Prediction errors for the machine learning estimates of the 2D projections of the plant bulb growth directions, shown in both (**a**) histogram and (**b**) scatter plot form. The Kernel ELM and ANN algorithms outperform Kernel SVR, and the errors of all 3 algorithms seem independent of the ground truth angles. This suggests that the algorithms are working in an unbiased manner.

Figure 3a shows the histograms of prediction errors for the estimation of the 2D growth angles. As expected, the prediction errors cluster around zero, though the ANN and kernel ELM algorithms generally produced lower errors than kernel SVR. Kernel ELM produced a larger percentage of estimates with less than 5° error, but its remaining errors were more spread out than those of the ANN. This effect is most notable in the error range between 5–15° where the ANN results outnumber kernel ELM. Figure 3b further shows a scatter plot of the prediction errors with respect to the ground truth 2D growth angles. Note that the prediction errors appear to be uniformly spread across all ground truth angles for all three algorithms. This suggests that the algorithms are unbiased to the ground truth growth angles. We note that the prediction errors do vary with respect to the flower bulb being tested, and that a small number of flower bulbs show significantly higher prediction errors than the majority of the dataset (see Supplementary Fig. S1).

### Full simulation

**Figure 4:**
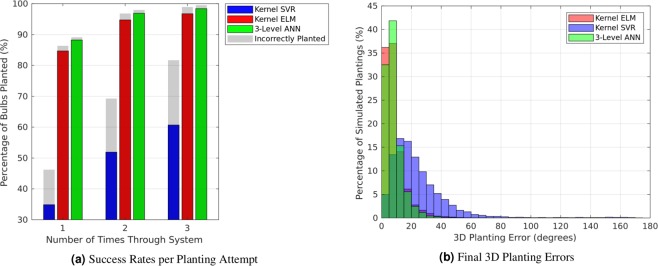
Final 3D growth direction planting errors for our simulation study, shown in both (**a**) success rate and (**b**) histogram form. After a maximum of 3 attempts to determine the flower bulb’s growth direction, 98.4% of the simulated flower bulbs can obtain a successful growth direction estimate (error < 30°) using the proposed 3-layer ANN.

Figure 4a shows, in colour, the percentage of flower bulb simulations that received a successful growth direction estimate (error < 30°), and in grey, the percentage that received a poor growth direction estimate (error $\ge \,{30}^{\circ }$). Results are shown cumulatively based on the number of times a bulb was run through the simulation. As expected from the 2D results, the ANN slightly outperformed the kernel ELM, while both algorithms significantly outperformed kernel SVR. After a maximum of three passes through the simulation, 98.40% of cases received in a successful growth direction estimate from the ANN, while kernel ELM and kernel SVR achieved success rates of 96.76% and 60.68%, respectively.

Figure 4b further shows the histogram of estimation errors for the growth directions in the cases where an estimate was outputted by the system. Note that the errors have increased over those estimated in 2D (see Fig. 3a), but that the general trends persist: the kernel ELM produces accurate results (error < 5°) for more cases than the other algorithms, but the ANN results generally cluster more tightly around zero. Both algorithms continue to outperform kernel SVR. We further observed that, for 66 of the 68 flower bulbs, the system provided a successful estimate over 90% of the time using the ANN. The remaining two bulbs, while obtaining relatively worse results, still obtained a successful estimate over 80% of the time (see Supplementary Fig. S2).

**Figure 5:**
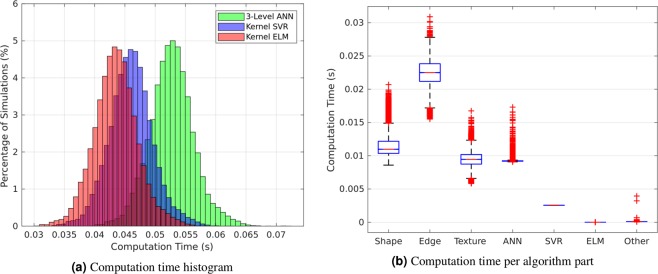
Computation time results for the growth direction estimation algorithm, shown both (**a**) cumulatively and (**b**) per step in the framework. Times are reported as the number of seconds per flower bulb. Note that each flower bulb receives an estimate within less than 0.1 seconds regardless of machine learning algorithm used. This allows us to achieve the industry criteria of 10 estimates per second in each case.

Finally, Fig. 5a shows the histogram of computation times for each of the 27,200 simulations performed in this study. In all cases and for all machine learning algorithms, an estimate was obtained in less than 0.1 seconds. The timings for each part of the system - the feature selection steps (shape, edge, and texture), the machine learning regressors, and everything else combined - is displayed in Fig. 5b. We observed that the edge features were the most computationally expensive part of the system, and that the ANN was the most computationally expensive machine learning algorithm. Nevertheless, all parts of the system are sufficiently fast to meet industry standards^22^.

## Discussion

**Figure 6:**
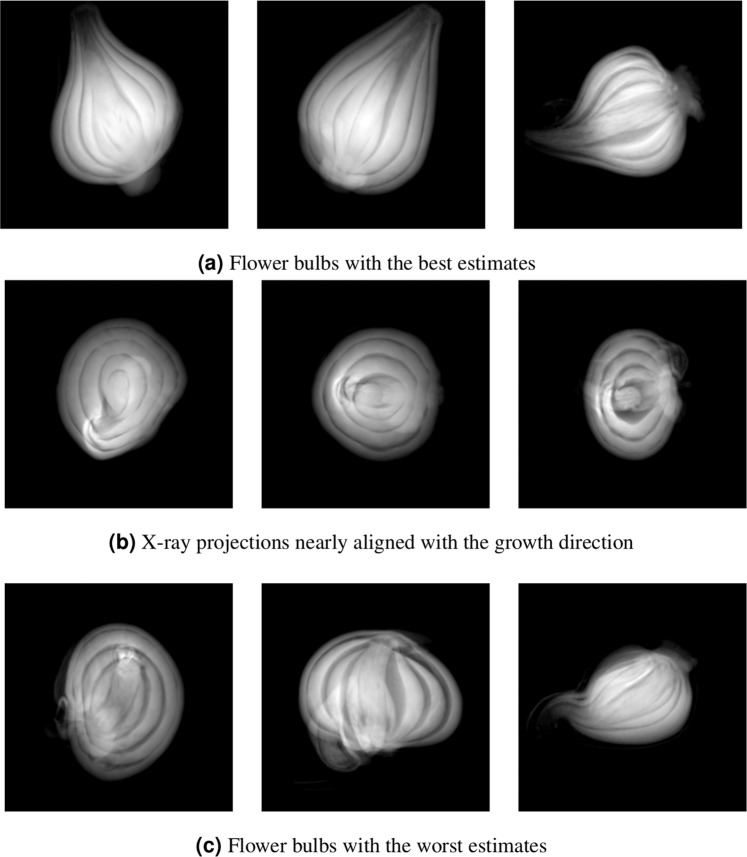
Examples of x-ray projection images from flower bulbs with both (**a**) good and (**b,c**) bad growth direction predictions from our framework. Note that the framework performs well when the bulbs are reasonably symmetric with clear shells and a noticeable tip. Worse results are seen when the bulbs have a curved stem, low contrast between shells, or have growths on the outside of the bulbs. Additionally, x-ray projection images where the normal of the projection plane is nearly in line with the growth direction of the flower bulb also proved challenging for the framework. See text for further details.

We have presented herein a machine learning framework for plant bulb growth direction estimation. The framework is based on shape, edge, and texture features collected from three non-colinear x-ray projections of the plant bulb. Using machine learning regressors and the geometric relationships between the x-ray projectors, we are able to obtain a 3D estimate of the plant bulb’s growth direction from a triplet of 2D x-ray images. One of the key aspects of the proposed framework is the filtering of 3D estimates that do not internally agree with each other. This filtering stage was introduced to account for the fact that certain x-ray projections may have been acquired from an angle where the plant bulb’s shape, edge, and texture features are uninformative. Figure 6b shows examples of such cases; they typically occur when the normal of the projection plane points in roughly the same direction as the bulb’s growth direction. It is also for this reason that we chose not to have the machine learning algorithms estimate the 3D growth angle directly from the three x-ray projections at once: Since any one of the three projections could be uninformative, the algorithms would have the additional challenge determining which of the projection images to ignore.

With respect to the three machine learning regression algorithms we evaluated, we obtained the best overall results with the 3-layer ANN with kernel ELM running a close second. This outcome may be due to two factors. First, the kernel ELM sets some of its network’s weights randomly as opposed to optimizing them. While this usually improves generalizability, it can also inflate errors due to the decreased flexibility in the regression function. Second, the additional layers in the ANN provide a function composition effect that can produce more powerful regressors than the single hidden layer network used in kernel ELM^47,48^. Finally, both kernel ELM and the ANN far outperformed kernel SVR, suggesting that this regression problem is a non-linear one even after applying a Gaussian kernel to the inputted image features.

We further noticed that the quality of our algorithm’s growth direction estimates depended on the plant bulb being imaged. As a result, we qualitatively examined the 3 plant bulbs that had the most successful growth direction estimates and the 3 bulbs with the least number of successful growth direction estimates. Those plant bulbs are shown in Fig. 6a,c, respectively. For the most successful bulbs, we note that the tip of the bulb is clearly visible and can be easily captured by the shape features. Also, the bulb is rather symmetric and has good contrast between its shells and the space between them, allowing for our edge features to easily identify the bulb’s central shaft. This is not the case for the less successful bulbs, where a variety of visual challenges appear. These challenges include a bent central shaft (all three images in Fig. 6c), low contrast between the bulb’s shells (right image in Fig. 6c), and asymmetries in the bulb’s shape, in some cases due to the presence of bulblets along the side of the bulb (centre image in Fig. 6c, lower left corner of the bulb). These cases were rare in our database and it is possible that including additional bulbs like these in the training set could improve the performance of the proposed framework. Training and testing with more plant bulbs is something we intend to pursue as future work.

Overall, the proposed framework was able to achieve the industry-desired timing criteria (0.1 seconds) in all cases, and achieved the accuracy criteria (error < 30°) in up to 98.40% of cases using the ANN. These simulation results on 27,200 cases indicate that the proposed algorithm is reliable enough to proceed with evaluation as part of a physical robotic bulb planting prototype. This move from a simulation to a physical prototype may introduce additional challenges such as the inclusion of the conveyor belt within the image and additional background noise. We hypothesize that these additional challenges might be overcome with the addition of standard image processing techniques: the conveyor belt could be subtracted from the image using a template image acquired in the absence of a plant bulb^49^, and additional noise could be addressed using image filtering techniques^50^. These additional computational steps may increase the overall computation time. That being said, the use of unoptimized MATLAB code in this simulation study indicates that opportunities remain to improve the speed of the current algorithm in order to accommodate these additional image processing steps.

In conclusion, we have proposed that the automated planting of bulbous plants introduces the unique computer vision task of growth direction finding. To address this task, we have introduced the first algorithm for automated plant bulb growth direction finding. The algorithm makes use of machine learning regressors and visual features from x-ray projections of the bulbs in order to estimate the bulbs’ projected 2D growth directions. These 2D estimates are then combined from image pairs and mapped to the 3D world coordinate system. Finally, estimates from multiple image pairs are compared to each other in order to determine the quality of the estimate, with poor quality estimates being discarded. Our results on a simulation of 27,200 cases resulted in successful estimates 98.40% of the time, suggesting that it is worthwhile to extend this algorithm to testing in a physical prototype.

## Methods

An overview of our growth direction estimation algorithm was shown in Fig. 1 and consists of four main components: feature selection, 2D angle prediction, the 2D-to-3D mapping, and the filtering out of plant bulbs whose estimates disagree with each other. Each of these algorithmic components are presented in detail below.

### Feature selection

The first step in the growth direction estimation algorithm is to extract a small set of features from the x-ray projection images that can simplify the regression step between the images and the corresponding growth directions. We identify three such features of interest below.

#### Shape features

The shape of the plant bulb is characterized by comparing it to the shape of an ellipse of the same area, positioned at the same location and orientation in the image as the bulb itself. In this way, we highlight the non-elliptical features of the bulb shape, particularly the bulb’s tip, for the growth direction estimation.

We obtain the shape of the plant bulb by thresholding the image at the empirically-chosen threshold of 5% of the image’s maximum intensity, resulting in a binary segmentation ${S}_{bulb}$. From this resulting segmentation, a principal component analysis is performed on the pixel locations within the plant bulb (i.e. $\{(x,y)|{S}_{bulb}(x,y)=1\}$) in order to obtain the bulb’s centroid $({x}_{c},{y}_{c})$ and major/minor axes of variation. These pixel locations are then projected onto the two axes of variation to obtain the length and width of the bulb $(a,b)$. This information is then used to define a comparable ellipse segmentation ${S}_{ellipse}$,

1 $${S}_{ellipse}(x,y)=(\begin{array}{ll}1 & {\rm{if}}\,\frac{{(x\cos (\alpha )+y\sin (\alpha )-{x}_{c})}^{2}}{{a}^{2}}+\frac{{(x\sin (\alpha )-y\cos (\alpha )-{y}_{c})}^{2}}{{b}^{2}}\le 1\\ 0 & {\rm{o}}{\rm{t}}{\rm{h}}{\rm{e}}{\rm{r}}{\rm{w}}{\rm{i}}{\rm{s}}{\rm{e}}\,\end{array},$$

This ellipse segmentation is subtracted from the plant bulb segmentation in order to emphasize the non-elliptical elements of the plant bulb’s shape: ${S}_{diff}=S-{S}_{ellipse}$.

**Figure 7:**
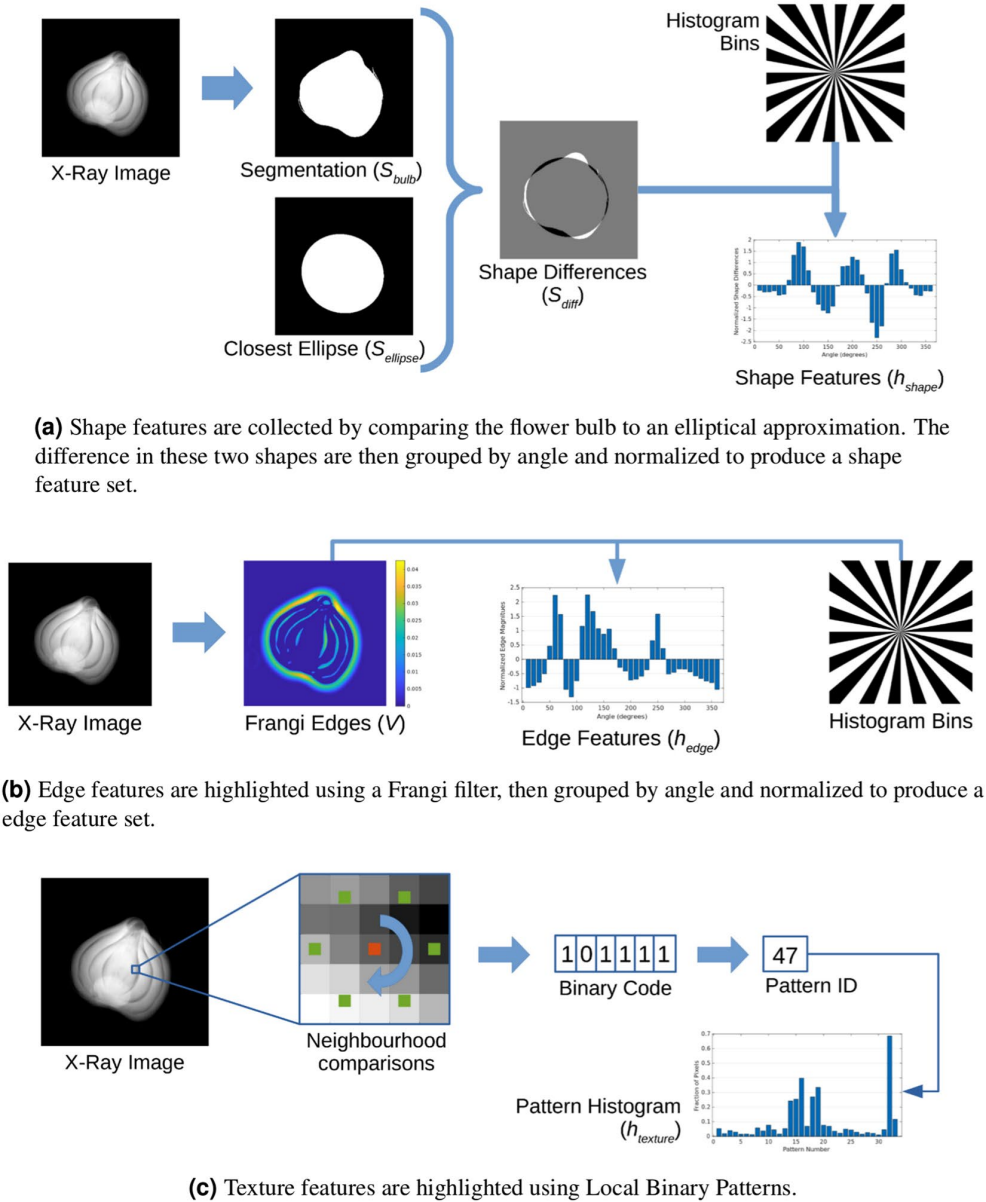
Image features used for growth angle estimation include (**a**) shape, (**b**) edge, and (**c**) texture information. See text for further details.

Additionally, we desire to reduce the dimensionality of the shape information – and make it invariant to the bulb’s position in the image – in order to aid the subsequent neural network regression. To achieve this goal, we bin the resulting segmentation differences into an angular histogram. The angular histogram *h*_*shape*_ is defined as being centred at the centroid of the plant bulb segmentation $({x}_{c},{y}_{c})$ and has each bin covering 10°. Finally, to make the histogram invariant to plant bulb size, we statistically normalize the histogram elements by their z-scores. The resulting normalized histogram *h*_*shape*_, containing 36 entries, is then taken as the final set of shape features for the x-ray projection image. A summary of the shape feature definition is shown in Fig. 7a.

#### Edge features

Another notable visual feature in x-ray projections of plant bulbs is the pattern of shell-shaped structures that compose the majority of the bulb’s mass. These structures are denoted as scales and contain nutrients for the plant^15^. These scales surround the shoot and the bulb’s growth direction, which makes them a valuable image feature for the estimation of the bulb’s growth direction.

To capture this visual feature, we apply the vesselness filter of Frangi *et al*. to the given x-ray projection image^28^. Originally designed to highlight blood vessels in angiogram images, the Frangi vesselness filter works to highlight layered or tubular structures, structures similar in appearance to the shells in our x-ray projection images. The Frangi vesselness filter produces an edge image, *V*, defined as

2 $$V(x,y)=(\begin{array}{ll}exp[-\frac{{\lambda }_{2}^{2}}{2{\lambda }_{1}^{2}{\beta }^{2}}](1-exp[-\frac{{\lambda }_{1}^{2}{\lambda }_{2}^{2}}{2{c}^{2}}]) & {\rm{if}}\,{\lambda }_{1} < 0\\ 0 & {\rm{otherwise}}\,\end{array},$$

where $\beta =0.5$ and *c* = 15 control the influence of the “blobness” and “structure” terms respectively. Both terms are based on the eigenvalues, $|{\lambda }_{1}|\le |{\lambda }_{2}|$, of the image’s Hessian matrix. The blobness term encourages one eigenvalue to be larger than the other, thereby highlighting areas where the image gradient is strong only in one direction. The structure term encourages the pair of eigenvalues to be large, thereby highlighting only areas where we see clear intensity changes. We ignore cases where ${\lambda }_{1} > 0$ so as to only highlight high-intensity structures, which is how plant bulb shells appear in x-ray projection images. To capture shells of different thicknesses, we apply the Frangi filter at multiple scales by convolving the image with Gaussian filters of $\sigma =\{3,5,7\}$ prior to computing the Hessian^28,29^. The scale that produces the greatest filtered response is then retained in the edge image *V*.

As with the shape features, we summarize this edge information in a statistically-normalized angular histogram. The resulting normalized histogram, ${h}_{edge}$ contains 36 elements and is taken to be our final set of edge features. A summary of this feature detection technique is shown in Fig. 7b.

#### Texture features

While the previous feature detectors identify the shape and edges of the plant bulb, they fail to capture more complicated visual textures within an image. It has been shown that adding image texture features to a machine learning task can improve its performance^51^ and so we also follow that convention here. To capture the texture features of the plant bulbs, we employ local binary patterns (LBP)^31^.

As the name suggests, LBP works at the level of individual pixels and generates a binary feature vector by comparing each pixel’s intensity to its neighbours. A pixel’s set of neighbours can be defined in many ways, but here we use the circular neighbourhood definition of Ojala *et al*.^30^. Given a user-chosen radius *r* and number of neighbours *k*, a pixel’s neighbours are defined as a set of *k* pixels uniformly spread around a circle of radius *r* centred at the chosen pixel (Fig. 7c). LBP then proceeds by comparing the intensity of a pixel to each of its neighbours. Wherever the intensity of the neighbour pixel is larger, a binary 1 is recorded for that neighbour. Otherwise, the neighbour is given the label 0. The result of these pixel comparisons are *k* binary digits which, when ordered in a clockwise manner, gives a *k*-digit binary number. Each binary number represents a different local pattern or texture. A summary of LBP is provided in Fig. 7c.

The LBP algorithm provides us with a binary number at each pixel, numbers which are then converted to decimal and binned into a histogram ${h}_{texture}$. In this work, we empirically set $r=2$ and $k=6$, which combined with the use of a global uniform pattern bin^30^, results in a histogram of 33 entries.

### Machine learning regression

Using the combination of shape, edge, and texture features described earlier, we predict the 2D angle associated with the plant bulb’s growth direction in the x-ray projection image. For this purpose, we evaluate three machine learning regression algorithms: kernel support vector regression (SVR)^32^, kernel extreme learning machines (ELM)^34^, and a 3-layer fully-connected artificial neural network (ANN)^33^. Gaussian kernels were used for the SVR and ELM algorithms with a kernel width of 500 being selected via a line search. The details of our ANN are presented in Table 1.

**Table 1:** Structure and parameters of the artificial neural network used to predict the 2D angle of a flower bulb growth direction from its corresponding x-ray image features.

Category	Item	Details
Structure	Input Layer	1 input layer: 105 nodes (36 shape, 36 edge, 33 texture)
	Hidden Layer(s)	3 hidden layers (60 nodes, 30 nodes, 15 nodes)
	Output Layer	1 output layer: 2 nodes (sine & cosine of growth angle)
	Transfer Function	Hyperbolic Tangent
Optimization	Performance Function	Mean-Squared Error (with projection to unit circle)
	Backpropagation Algorithm	Scaled Conjugate Gradient (Hessian step size, σ: 5e-5) (Hessian regularization, *λ*: 5e-7)
	Maximum Number of Epochs	1000
	Gradient Tolerance	1e-06

A single input consisted of the 36 shape features ${h}_{shape}$, 36 edge features ${h}_{edge}$, and 33 texture features ${h}_{texture}$ described earlier. Each regressor’s output is the 2D angle $\theta$ of the plant bulb’s growth direction represented by its sine and cosine values. The sine and cosine of the angle are used to properly model the growth angle’s periodicity^52^. The predicted growth angle is then retrieved as $\theta ={\rm{atan}}2(\cos (\theta ),\,\sin (\theta ))$.

### 2D-3D geometric mapping

From the ANN, three 2D growth angles - ${\theta }_{1},{\theta }_{2},{\theta }_{3}$ - are predicted, one for each of the x-ray projection images. At this point in the algorithm, it is unclear whether all three angle predictions are accurate. As a result, we choose to introduce redundancy into the algorithm by converting pairs of 2D growth angles into 3D estimates of the plant bulb growth direction. In this fashion, we obtain three 3D growth direction estimates, one for each pair of predicted 2D growth angles (${\theta }_{1}$ and ${\theta }_{2}$, ${\theta }_{1}$ and ${\theta }_{3}$, ${\theta }_{2}$ and ${\theta }_{3}$).

To convert a pair of 2D growth angles into a single 3D estimate, we require information on the x-ray detector geometry. Let ${{\bf{x}}}_{i}$ be the 3D world coordinate defining the origin of the detector plane for x-ray image ${I}_{i}$. Furthermore, let ${{\bf{u}}}_{i}^{x}$ and ${{\bf{u}}}_{i}^{y}$ be vectors defining, in 3D world coordinates, the x-axis and y-axis of that detector plane, respectively. Their cross product ${{\bf{n}}}_{i}={{\bf{u}}}_{i}^{x}\times {{\bf{u}}}_{i}^{y}$ defines the normal of the detector plane in the direction of the x-ray source.

Given this detector geometry and a 2D growth angle ${\theta }_{i}$, we project outwards from the detector plane to identify the space to which the 3D plant bulb’s growth direction can belong. First, we convert ${\theta }_{i}$ to a vector representation:

3 $${{\bf{v}}}_{\theta ,i}=\,\cos (\theta ){\bf{u}}{}_{i}{}^{x}+\,\sin (\theta ){{\bf{u}}}_{i}^{y}+{{\bf{x}}}_{i}.$$

In this formulation, we define the vector as originating at ${{\bf{x}}}_{i}$, though any other point on the detector plane could be used since only the direction of the growth angle is of importance, not its exact location.

Since the 2D growth angle ${\theta }_{i}$ is parallel to ${{\bf{v}}}_{\theta ,i}$, we know that the corresponding 3D growth angle must also be parallel to a plane spanned by ${{\bf{v}}}_{\theta ,i}$ and the normal of the detector plane ${{\bf{n}}}_{i}$. We define this plane as a solution space $S(i)$ and parameterise it by its normal ${n}_{S(i)}={{\bf{v}}}_{\theta ,i}\times {{\bf{n}}}_{i}$. This process can similarly be done for the other x-ray projection.

Given two 2D growth angles, ${\theta }_{i}$ and ${\theta }_{j}$, we compute the normals of their solution spaces ${n}_{S(i)}$ and ${n}_{S(j)}$ respectively. We know that, since the 3D growth direction must lie both in $S(i)$ and $S(j)$, it must be orthogonal to both ${n}_{S(i)}$ and ${n}_{S(j)}$. Therefore, we define the 3D growth direction estimate, $({\theta }_{i,j},{\phi }_{i,j})$ as the azimuth and elevation angles defined by the cross product of these two normals:

4 $${\theta }_{i,j}={\cos }^{-1}(\frac{{n}_{S(i),x}{n}_{S(j),y}-{n}_{S(i),y}{n}_{S(j),x}}{\parallel {n}_{S(i)}\times {n}_{S(j)}{\parallel }_{2}}),\,{\phi }_{i,j}={\tan }^{-1}(\frac{{n}_{S(i),z}{n}_{S(j),x}-{n}_{S(i),x}{n}_{S(j),z}}{{n}_{S(i),y}{n}_{S(j),z}-{n}_{S(i),z}{n}_{S(j),y}}).$$

### Filtering of poor estimates

Following the pairwise 3D growth direction estimation step, we have three growth direction estimates: $({\theta }_{1,2},{\phi }_{1,2})$, $({\theta }_{1,3},{\phi }_{1,3})$, and $({\theta }_{2,3},{\phi }_{2,3})$. We hypothesize that if these three estimates agree with each other, then they are likely to be accurate. To measure the agreement between these angles, we compute the angular distance between each pair of angles as

5 $$d({\theta }_{i,j},{\phi }_{i,j},{\theta }_{a,b},{\phi }_{a,b})=\,\cos \,{}^{-1}[\sin \,({\theta }_{i,j})\sin \,({\theta }_{a,b})+\,\cos \,({\theta }_{i,j})\cos \,({\theta }_{a,b})\cos \,({\phi }_{i,j}-{\phi }_{a,b})]$$

These angular distances are then averaged across all three estimate pairs to obtain $\bar{d}$: a single measure of overall agreement between the growth direction estimates. A threshold $\tau$ is then applied to $\bar{d}$ to determine if the estimates agree with each other. If $\bar{d} < \tau$, then the three growth directions are assumed to be accurate and the average of the three growth direction estimates is outputted; if $\bar{d}\ge \tau$, then the plant bulb is imaged again. In this work, we empirically set $\tau ={40}^{\circ }$.

## Supplementary information

Supplementary information.
