# Supplementary material for: A Machine Learning Approach to Growth Direction Finding for Automated Planting of Bulbous Plants
Source: Sci Rep. 2020 Jan 20;10:661. doi: 10.1038/s41598-019-57405-8 (PMC6971015; doi:10.1038/s41598-019-57405-8)
Supplement: Supplementary file 1 — Supplementary information. [file 41598_2019_57405_MOESM1_ESM.pdf]

A Machine Learning Approach to Growth Direction Finding for Automated Planting of Bulbous Plants

Supplementary Material

Brian G. Booth, Jan Sijbers, and Jan De Beenhouwer
imec-Vision Lab, Department of Physics, University of Antwerp, B-2610 Antwerp, Belgium
brian.booth@uantwerpen.be

December 2, 2019

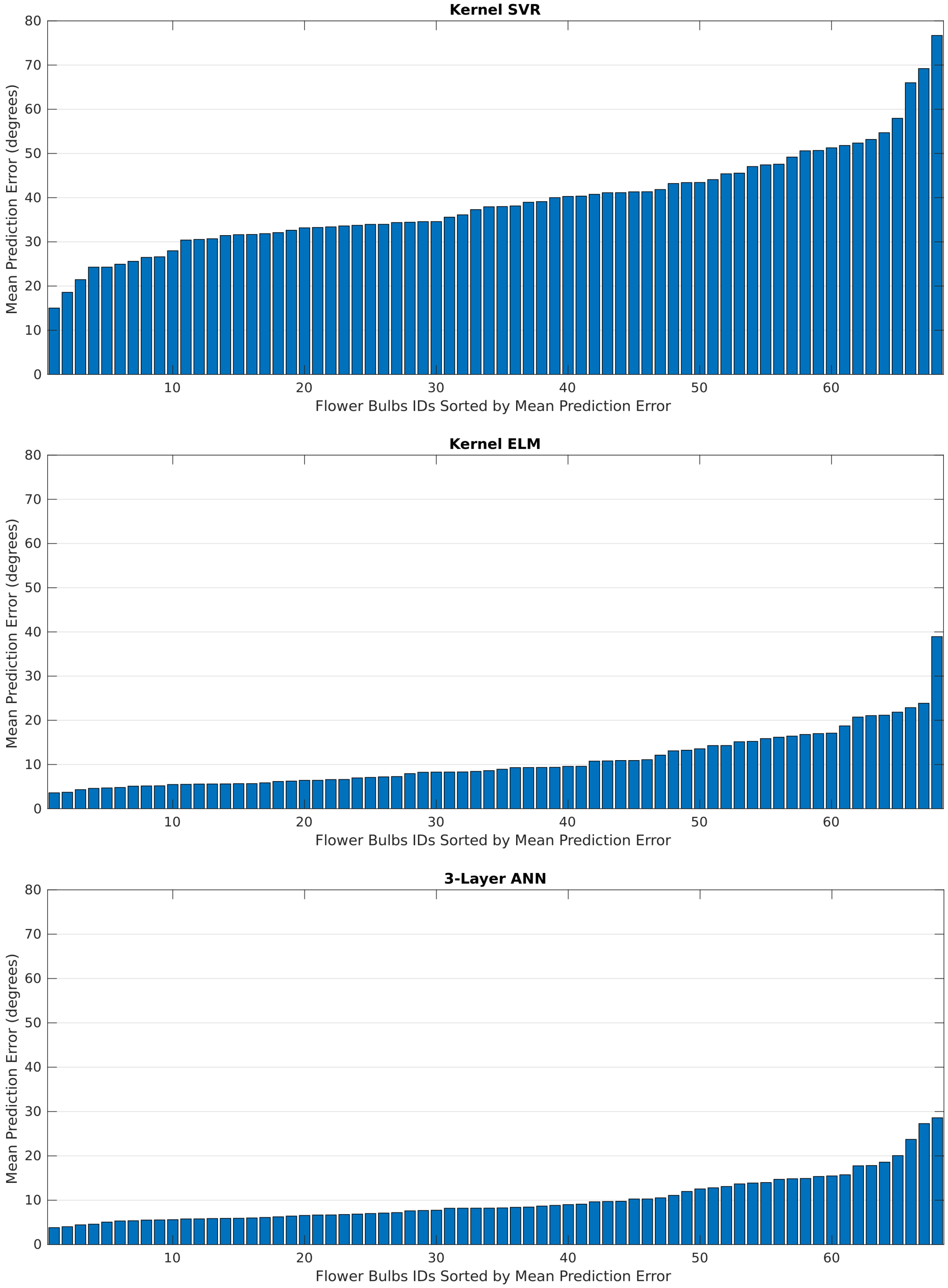

Figure 1: Mean 2D prediction error for each flower bulb in our database, averaged over 1200 test images per bulb. Note that some flower bulbs are outliers, suggesting that the machine learning algorithms could be improved by introducing more training data similar to the outlier bulbs.

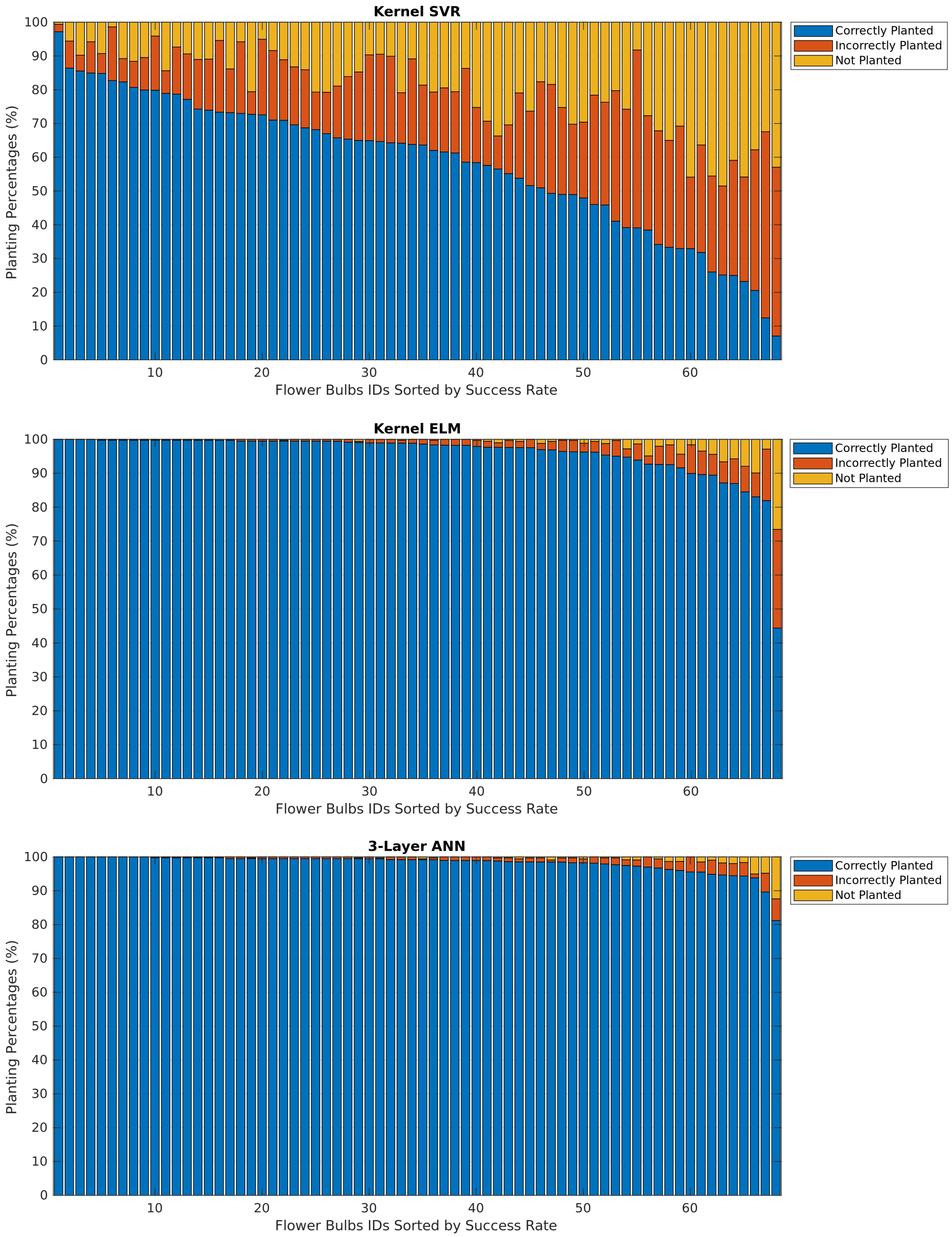

Figure 2: Growth direction estimation success rates for each flower bulb in our database, obtained from 400 conveyor belt simulations per bulb. Note that for the 3-Layer ANN, all flower bulbs received successful estimates at least 80% of the time, and for all but two of the bulbs, successful estimates were obtained at least 90% of the time.
